# The Cording Phenotype of *Mycobacterium tuberculosis* Induces the Formation of Extracellular Traps in Human Macrophages

**DOI:** 10.3389/fcimb.2017.00278

**Published:** 2017-06-26

**Authors:** Sadaf Kalsum, Clara Braian, Valerie A. C. M. Koeken, Johanna Raffetseder, Margaretha Lindroth, Reinout van Crevel, Maria Lerm

**Affiliations:** ^1^Division of Microbiology and Molecular Medicine, Department of Clinical and Experimental Medicine, Faculty of Health Sciences, Linköping UniversityLinköping, Sweden; ^2^Department of Internal Medicine and Radboud Center for Infectious Diseases, Radboud University Medical CenterNijmegen, Netherlands

**Keywords:** *Mycobacterium tuberculosis*, macrophage extracellular traps (METs), cording, Tween-80, virulence, early secreted antigenic target-6 (ESAT-6)

## Abstract

The causative agent of tuberculosis, *Mycobacterium tuberculosis*, shares several characteristics with organisms that produce biofilms during infections. One of these is the ability to form tight bundles also known as cords. However, little is known of the physiological relevance of the cording phenotype. In this study, we investigated whether cord-forming *M. tuberculosis* induce the formation of macrophage extracellular traps (METs) in human monocyte-derived macrophages. Macrophages have previously been shown to produce extracellular traps in response to various stimuli. We optimized bacterial culturing conditions that favored the formation of the cord-forming phenotype as verified by scanning electron microscopy. Microscopy analysis of METs formation during experimental infection of macrophages with *M. tuberculosis* revealed that cord-forming *M. tuberculosis* induced significantly more METs compared to the non-cording phenotype. Deletion of early secreted antigenic target-6 which is an important virulence factor of *M. tuberculosis*, abrogated the ability of the bacteria to induce METs. The release of extracellular DNA from host cells during infection may represent a defense mechanism against pathogens that are difficult to internalize, including cord-forming *M. tuberculosis*.

## Introduction

*Mycobacterium tuberculosis* is the causative agent of tuberculosis (TB), which is one of the world's deadliest diseases. The mechanisms that *M. tuberculosis* utilizes for intracellular growth are well-studied (Welin et al., 2008, 2011; Abramovitch et al., 2011), however, the pathology caused by its extracellular growth is poorly defined.

Mycobacteria, grown in a detergent-free liquid medium, appear as tight bundles or intertwined serpentine cords under the microscope (Middlebrook et al., 1947; Leisching et al., 2016). Moreover, scanning electron microscopy reveals the ultrastructure of mycobacterial cords, in which the orientation of the long axis of each cell is parallel to the long axis of the cord (Julian et al., 2010). The cord formation of mycobacteria has been shown to correlate with their virulence in a mouse model (Middlebrook et al., 1947; Glickman et al., 2000). However, the complex interplay between this virulence mechanism of the infecting pathogen and the immune response remains poorly understood.

Alveolar macrophages are one of the first host cell types to encounter *M. tuberculosis* in the lungs following aerosol transmission. Macrophages are well-known for their ability to eliminate various pathogens through phagocytosis, but recently, a phagocytosis-independent antimicrobial mechanism, referred to as extracellular traps (ETs), has been described in innate immune cells including macrophages (von Kockritz-Blickwede et al., 2008; Chow et al., 2010; Branzk et al., 2014) and also in social amoeba (Zhang et al., 2016). Neutrophil extracellular traps (NETs) have been widely studied in response to infections but also in a variety of other diseases (Malachowa et al., 2016). NETs have some bactericidal effects, but have also been shown to cause tissue damage (Papayannopoulos and Zychlinsky, 2009; Remijsen et al., 2011). These beneficial and detrimental effects can be assigned to the proteases and histones associated with the NETs. Macrophage extracellular traps (METs) are composed of double stranded DNA and histones and can be induced by many different microbes and microbial-derived toxins (Aulik et al., 2010; Wong and Jacobs, 2013; Liu et al., 2014). However, the mechanism that underlies MET formation is still obscure.

A recent study showed that extracellular traps from neutrophils are produced in response to large pathogens, such as *Candida albicans* hyphae and extracellular aggregates of *M. bovis*, but not in response to small yeasts or single bacteria, suggesting that neutrophils can sense microbe size and selectively produce NETs (Branzk et al., 2014). Furthermore, it has been demonstrated that non-sonicated bacteria induce more METs than sonicated bacteria, which indicates that mycobacterial clumps are more efficient at promoting extracellular traps (Wong and Jacobs, 2013). Additionally, cord-forming *M. abscessus*, a rapidly growing opportunistic mycobacterial species, induces the release of DNA meshwork by human peripheral blood mononuclear cells (PBMCs), a phenomenon which is not observed with non-cording strains (Jonsson et al., 2013). Therefore, the formation of ETs might be a defense mechanism against microbes that are too big to be ingested by innate immune cells.

The aim of this study was to examine the interaction between human monocyte-derived macrophages (hMDMs) and cord-forming *M. tuberculosis* and the ability of cording mycobacteria to induce METs in hMDMs. We observed that cording occurs when mycobacteria are grown under well-oxygenated conditions (shaken cultures) and with a reduced concentration of the detergent Tween-80 in the liquid medium. We believe that these conditions may mimic the *in vivo* milieu when mycobacteria grow in the air/fluid interface in lung cavities. The cord-forming *M. tuberculosis* were able to induce METs in hMDMs in an Early Secreted Antigenic Target 6 (ESAT-6)-dependent manner, which is a major virulence factor of *M. tuberculosis*.

## Methods

### Preparation of human monocyte-derived macrophages (hMDMs)

Peripheral blood mononuclear cells (PBMCs) were isolated from buffy coats obtained from healthy volunteers (Linköping University Hospital blood bank, Linköping, Sweden) as described elsewhere (Raffetseder et al., 2014). Healthy volunteers gave their written informed consent for the use of their blood for scientific purposes. PBMC isolation was performed using LymphoPrep (Axis-Shield), involving separation by a density gradient followed by multiple centrifugation and wash steps. The mononuclear cells were seeded in culture flasks in Dulbecco's Modified Eagle Medium (DMEM, Gibco), supplemented with 25 mM HEPES (Gibco), 100 U/ml penicillin (Gibco) and 100 μg/ml streptomycin (Gibco), and allowed to adhere for 2 h before the non-adherent lymphocytes were washed away. The adherent monocytes were allowed to differentiate into hMDMs at 37°C for 5–7 days in DMEM, containing 25 mM HEPES, 100 U/ml penicillin, 100 μg/ml streptomycin, 2 mM L-Glutamine (Gibco) and 10% active human serum pooled from 5 donors (Linköping University Hospital). The resulting macrophages have purity exceeding 99% (Raffetseder et al., 2014). One day prior to the experiment, cells were trypsinized and re-seeded in antibiotic-free medium in a 24-well plate with 0.13 mm glass coverslips (2.5 × 10^5^ cells/well).

### Bacterial strains and cultures

The virulent *M. tuberculosis* strain H37Rv (American Type Culture Collection) harboring the pFPV2-plasmid encoding green fluorescent protein (GFP) or pCherry3 plasmid carrying m-Cherry gene or pSMT1 plasmid for luciferase expression, an ESAT-6 deletion mutant strain (H37Rv-ΔESAT-6-GFP) and *Mycobacterium marinum* also harboring the pFPV2-GFP plasmid were used in this study (Valdivia et al., 1996; Snewin et al., 1999; Guinn et al., 2004; Carroll et al., 2010). Five additional *M. tuberculosis* strains, all belonging to lineage 1 (Nebenzahl-Guimaraes et al., 2016), were derived from the reference database of clinical isolates of the National Institute for Public Health and the Environment (RIVM) in Bilthoven, the Netherlands. These strains were selected from a study by Nebenzahl-Guimaraes et al. (2016), in which the strains were epidemiologically proven to be at the extremes of the transmission spectrum. The clinical strains 201 030 and 400 731 were both not part of a cluster, while 500 395, 500 887, and 800 343 all showed a high level of clustering, meaning that these strains were able to cause multiple subsequent TB cases. These five clinical isolates were transformed with the pFPV2-GFP plasmid.

All strains were grown in Middlebrook 7H9 broth supplemented with albumin-dextrose-catalase (ADC, Becton-Dickinson), and 0.05% Tween-80 for 2–3 weeks at 37°C. For standing cultures, the bacteria were re-seeded in fresh broth in the presence of 0.05% Tween-80 (Tween_high_) for an additional 7 days to reach log phase. The shaken cultures were passaged 3 days prior to the experiment in medium without Tween-80, leading to the final concentration of 0.01% (Tween_low_), and were put on a shaker at 260 rpm. Kanamycin at 20 μg/ml and hygromycin at 100 μg/ml were used as selection antibiotics where required. Prior to this study, standardization experiments were performed to test culture conditions, and determine the optimal duration of culturing (3 and 7 days) and shaking speed (260 rpm; data not shown).

### Experimental infection

On the day of infection, the bacteria were collected and prepared as previously described (Eklund et al., 2010). In brief, both bacterial suspensions of shaken and standing cultures were centrifuged at 5,000 × g for 5 min in phosphate-buffered saline (PBS) supplemented with 0.05% Tween-80 and passaged through a sterile syringe equipped with a 27-gauge needle to remove aggregates. This procedure was repeated once and the bacteria were resuspended in DMEM supplemented with 25 mM HEPES. The numbers of bacteria/ml was determined both for cording and non-cording conditions (the OD was validated against CFU for both situations, Figure S1) by using optical density at 600 nm (OD600) as a function of CFU/ml. The bacteria were added to the hMDMs at a multiplicity of infection (MOI) of 10. The macrophages were incubated with mycobacteria for 1 h at 37°C before medium was changed to DMEM supplemented with 25 mM HEPES and 10% active human serum pooled from 5 donors (from blood bank at Linköping University hospital).

### Confocal microscopy

To characterize METs, 2.5 × 10^5^ hMDMs were seeded in wells on glass coverslips and infected as described above. After 24 h of incubation, the cells were washed once with PBS and incubated with 180 U of DNase I (Thermofisher) for 30 min. To characterize dsDNA, the cells were stained with Quant-iT Picogreen dsDNA kit (Invitrogen) for 5 min. To determine the dependency of MET-formation on ROS-production, hMDMs were incubated with 10 μM of the NADPH-oxidase inhibitor Diphenylene iodonium (DPI) or only dimethyl sulfoxide (DMSO) as a solvent control, for 30 min before infection. DPI and its solvent DMSO were purchased from Sigma Aldrich (Saint Louis, MO). After all the above procedures, cells were washed twice with PBS before fixation in 4% paraformaldehyde (PFA) at RT for 30 min. After fixation, samples were stained with the nuclear dye DAPI, mounted in mounting medium (Dako) and visualized with a Zeiss LSM 700 upright confocal system.

### Immunostaining

For immunofluorescence staining, infected hMDMs (2.5 × 10^5^), seeded on glass coverslips, were fixed with 4% PFA, blocked (2% BSA and 10% goat serum in PBS) and incubated with polyclonal rabbit anti-histone H4 Citrulline 3 (diluted 1:50; Millipore) at RT for 1 h. After washing three times with PBS, samples were incubated with secondary Alexa 594-lableled goat anti-rabbit antibody (diluted 1:800; Molecular Probes, Eugene, OR) for additional 30 min at RT, again washed three times with PBS and stained with DAPI before mounting onto the slides.

### Scanning electron microscopy

hMDMs were seeded onto glass coverslips for 24 h before infection as described above. After 24 h of infection the cells were washed twice with 0.15 M cacodylate buffer and fixed with 2% glutaraldehyde for 1 h at RT. Subsequently, the samples were dehydrated with a graded ethanol series (50, 70, 85, and 100%), critical point dried with liquid CO_2_ and sputter-coated with 1.5 nm platinum. The samples were examined using a scanning electron microscope (JSM 6320F).

### Luminometry-based measurement of bacterial numbers

A previously set up luminometry-based method (Eklund et al., 2010) was used to compare the kinetic of mycobacterial growth harvested from either shaken or standing cultures during hMDMs infection. Aliquots of medium supernatant and cell lysate containing luciferase-expressing bacteria were transferred to pure water in white 96-well plates at selected time points. One percent of decanal (Sigma Aldrich) was injected through the instrument needle into each well and flash luminescence was measured using a Glomax Multiplus reader (Promega). Light emission, measured as arbitrary luminescence units (ALU), is linearly proportional to the number of viable bacilli. Both extracellular (supernatant) and intracellular (lysate) number of bacteria were obtained by subtracting the ALU values from uninfected cells. The ALU values from both supernatant and lysate were then standardized for dilutions and summed up to obtain total ALU values. To determine fold change of bacterial load, the median value of each triplicate of all time points was normalized to the initial time point of the same experiment.

### Quantification of extracellular DNA

For quantification of MET production, specimens of infected hMDMs were coded and analyzed blindly using a confocal microscope. Briefly, the total number of nuclei and the number of nuclei connected to METs were counted in eight individual images per sample, after which the mean MET production for each sample was calculated. Nuclei releasing longitudinal thread-like structures were considered as producing METs. For each sample, at least 700 nuclei were examined in total.

### Assessment of oxidative burst

Reactive oxygen species (ROS) production was measured using luminol-enhanced chemiluminescence (5-amino-2,3, dihydro-1,4-phtalazinedione, Sigma-Aldrich) in a plate reader (GloMax Multiplus reader, Promega). ROS oxidizes luminol to an intermediate form, luminophore, which is in an excited state and emits light with an emission peak at 425 nm. The intensity of the chemiluminescence emitted is proportional to the amount of ROS in the sample. Before infection, the hMDMs were washed once with 200 μl 0.5% bovine serum albumin (BSA) HBSS in a 96-well round-bottom plate. The cells were either activated with *M. tuberculosis* (MOI 10), zymosan (833 μg/ml) as a positive control, or culture medium alone. Subsequently, chemiluminescence was measured at 37°C for 100 min and as a measure of ROS production, the area under the curve (AUC x time) was determined.

### Statistical analysis

Statistical analysis was performed using the unpaired Student's *t*-test (for two groups) and one - way ANOVA (for more than two groups) with the GraphPad Prism 6 software. Data presented were expressed as median values ± SEM and *P*-values of 0.05 or less were considered statistically significant.

## Results

### Shaken H37Rv cultures form organized structures

**Figure 1:**
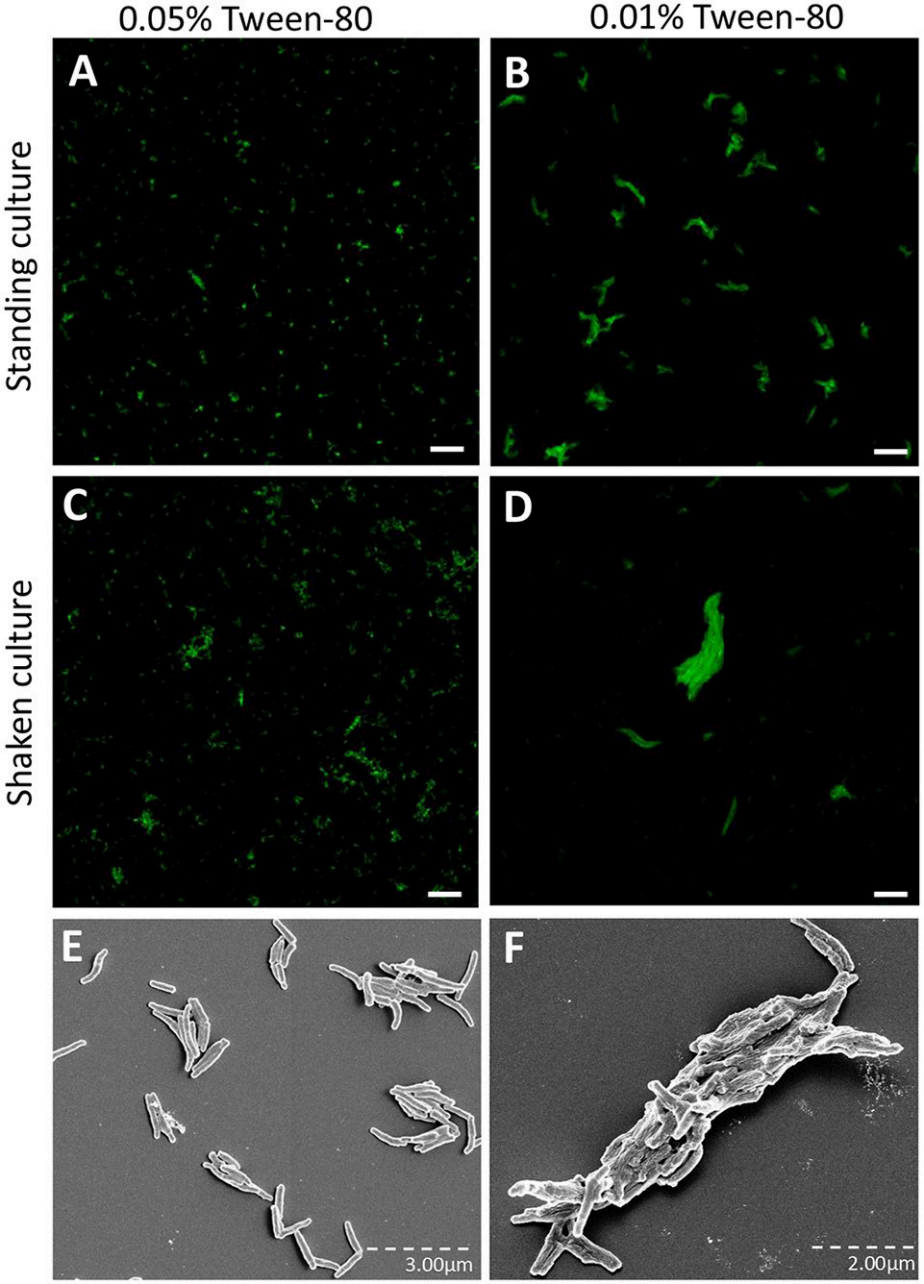
H37Rv cultured in broth with reduced Tween-80 concentration and on a shaker form cords. GFP-expressing H37Rv was passaged and cultured in the presence of **(A,C,E)** 0.05% Tween or **(B,D,F)** 0.01% Tween for either **(A,B,E)** 7 days as a standing culture or **(C,D,F)** for 3 days on a shaker at 260 rpm. The bacteria were prepared with multiple passages through a 27-gauge syringe, incubated on cover slips overnight and fixed. Images were taken through either **(A–D)** confocal microscopy or **(E,F)** scanning electron microscopy. Bar: 20 μm.

*M. tuberculosis* is routinely cultured in a liquid culture medium containing the detergent Tween-80 to avoid aggregation of the bacilli. As the cording phenotype represents a form of organized aggregate, we speculated that reducing the concentration of Tween-80 would promote cord formation. To test this, an H37Rv strain expressing GFP was cultured for 3 days on a shaker at 260 rpm in broth with either 0.01% Tween-80 (Tween_low_) or 0.05% Tween-80 (Tween_high_). These cultures were compared to standing cultures of *M. tuberculosis* with Tween_low_ or Tween_high_ broth for 7 days. Fluorescence microscopy analysis revealed that the standing cultures, especially in the Tween_high_ broth contained bacteria without any large aggregates (Figures 1A,C). In contrast, the shaken cultures formed larger, more organized aggregates, especially in the Tween_low_ broth (Figures 1B,D). Scanning electron microscopy performed on bacteria harvested from the two extreme culture conditions (standing cultures with Tween_high_ and shaken cultures with Tween_low_) revealed the presence of cords only in shaken cultures with Tween_low_ (Figures 1E,F). Based on this observation, we hereafter define the bacterial phenotypes as “cording” (shaken with Tween_low_) or “non-cording” (standing with Tween_high_).

### Cording H37Rv induces MET formation

**Figure 2:**
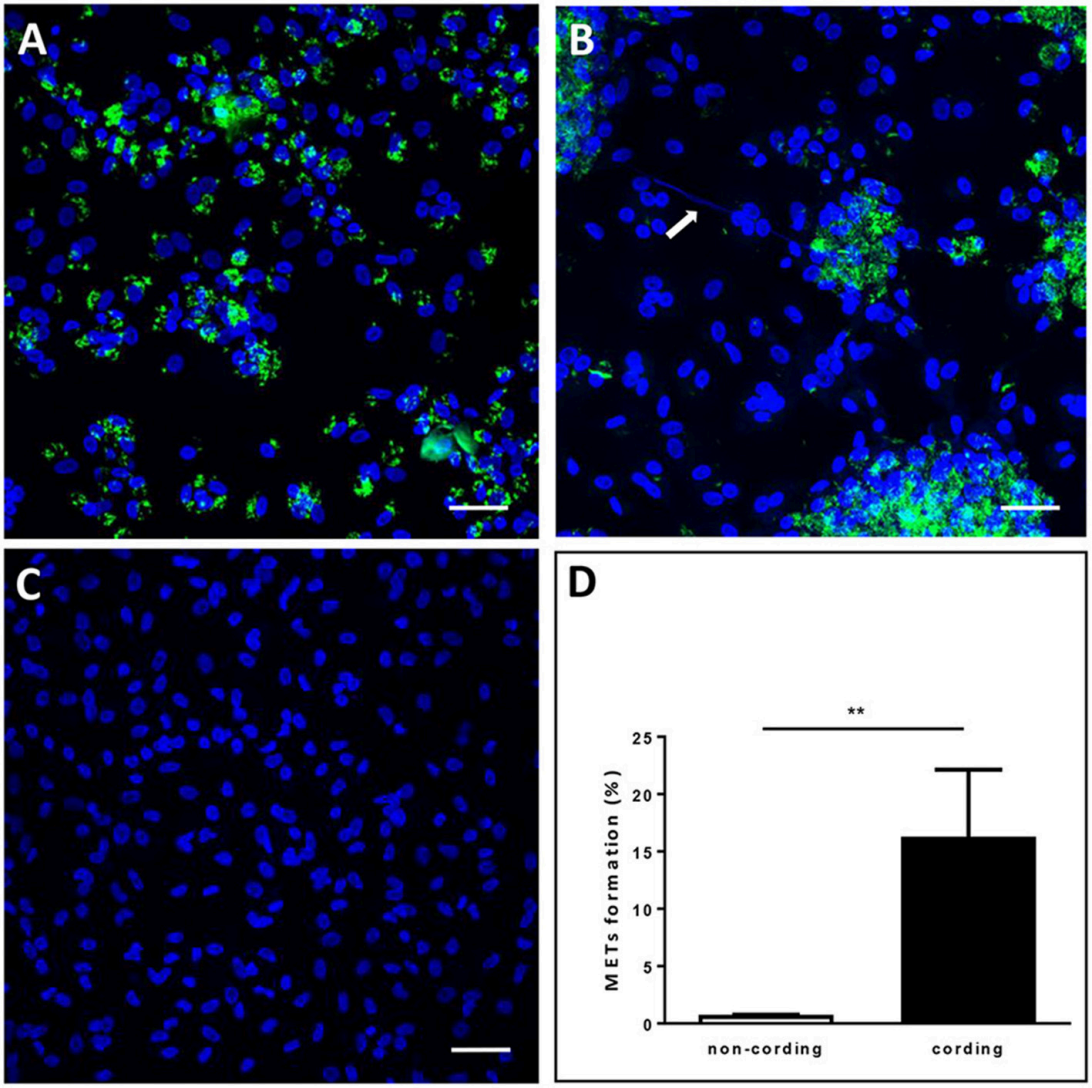
Cording *M. tuberculosis* induces MET formation in human macrophages. hMDMs were infected with GFP-expressing H37Rv (green) **(A)** non-cording or **(B)** cording at MOI 10 or **(C)** left uninfected. The cells were then incubated for 24 h, fixed and stained with DAPI (blue). The white arrow indicates METs release by human macrophages. Bar: 20 μm. **(D)** The percentage of MET-forming macrophages was quantified after 24 h of H37Rv infection. Mean values ± SEM from 8 images are shown (*n* = 3). ^**^ *p* < 0.01.

**Figure 3:**
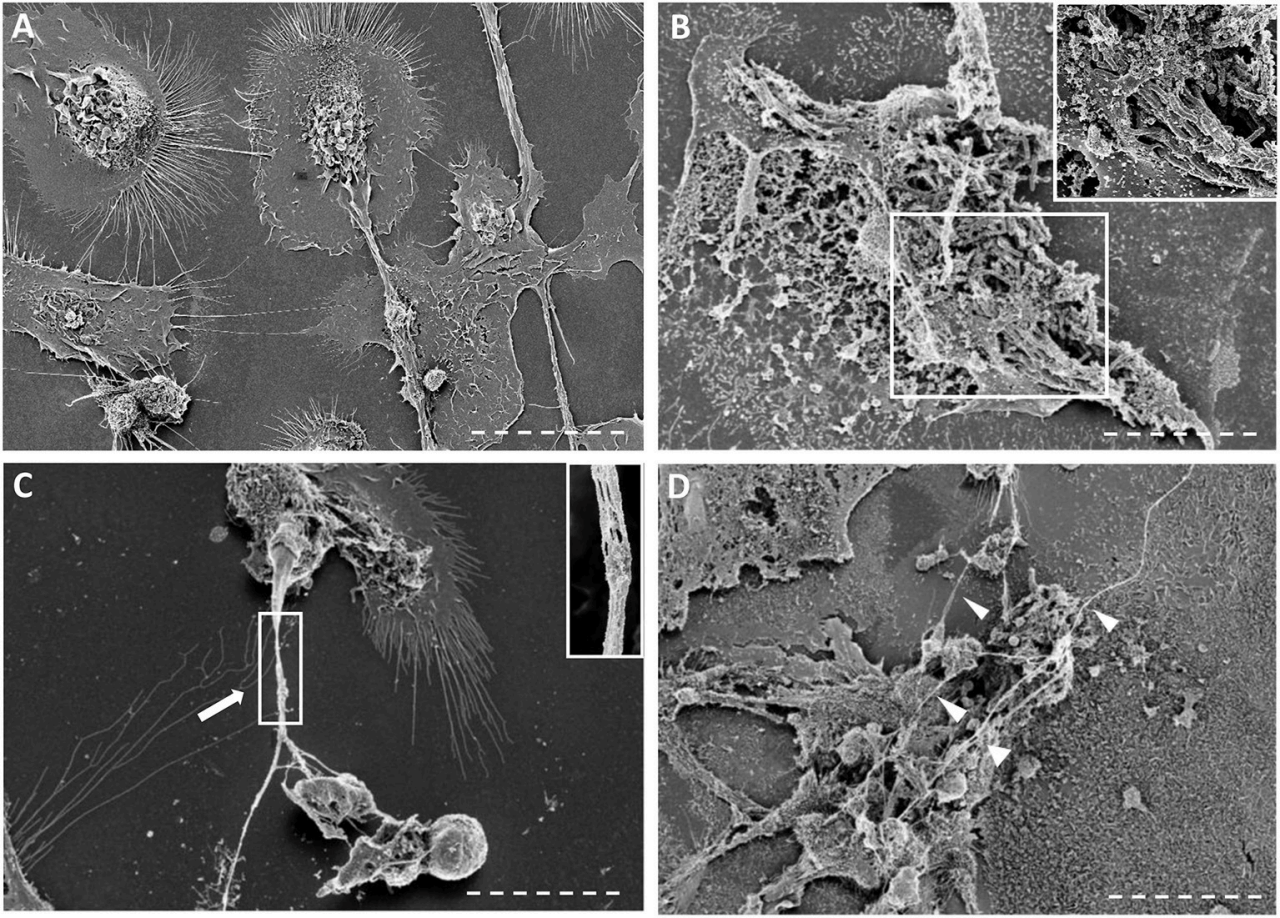
Scanning electron microscopy images of macrophages infected with non-cording or cording cultures. **(A–D)** Scanning micrographs of cells fixed with glutaraldehyde after 24 h of infection either with **(A)** non-cording bacteria or **(B–D)** cording bacteria. METs formed by human macrophages are either **(C)** in form of threads or **(D)** meshwork. The white arrows indicate the thread of DNA while the arrow heads show the meshwork. Bar: 10 μm

Next, we assessed whether cording *M. tuberculosis* can induce MET formation in hMDMs. We infected hMDMs with the GFP-expressing cording or non-cording phenotypes of H37Rv. Uninfected cells were included as a control. DAPI-staining of DNA revealed that cording bacteria induced the release of extracellular DNA in hMDMs (Figure 2B), while macrophages infected with non-cording bacteria produced relatively few METs (Figure 2A). In uninfected cells, no METs could be observed (Figure 2C). Kinetic analyses at 2, 4, 6, and 24 h showed that cording appeared from 6 h on (not shown). Also, with increasing MOIs of the standing culture no METs were observed (Figure S2). Quantification of the percentage of hMDMs producing METs confirmed the observation that infection with the cording phenotype induced significantly more METs (17.0%) compared to non-cording bacteria (1.0%; Figure 2D). Next, we used scanning electron microscopy to observe the ultrastructure of METs (Figures 3A–D). Human macrophages infected with the non-cording bacteria showed normal morphology even after 24 h of infection (Figure 3A). On the other hand, mycobacterial cords could be seen in the remnants of dead macrophages infected with the cording phenotype (Figure 3B) and the macrophages had released extracellular traps either in the form of threads (Figure 3C) or meshwork (Figure 3D).

### METs triggered by cording H37Rv are composed of DNA and histones and independent of ROS

**Figure 4:**
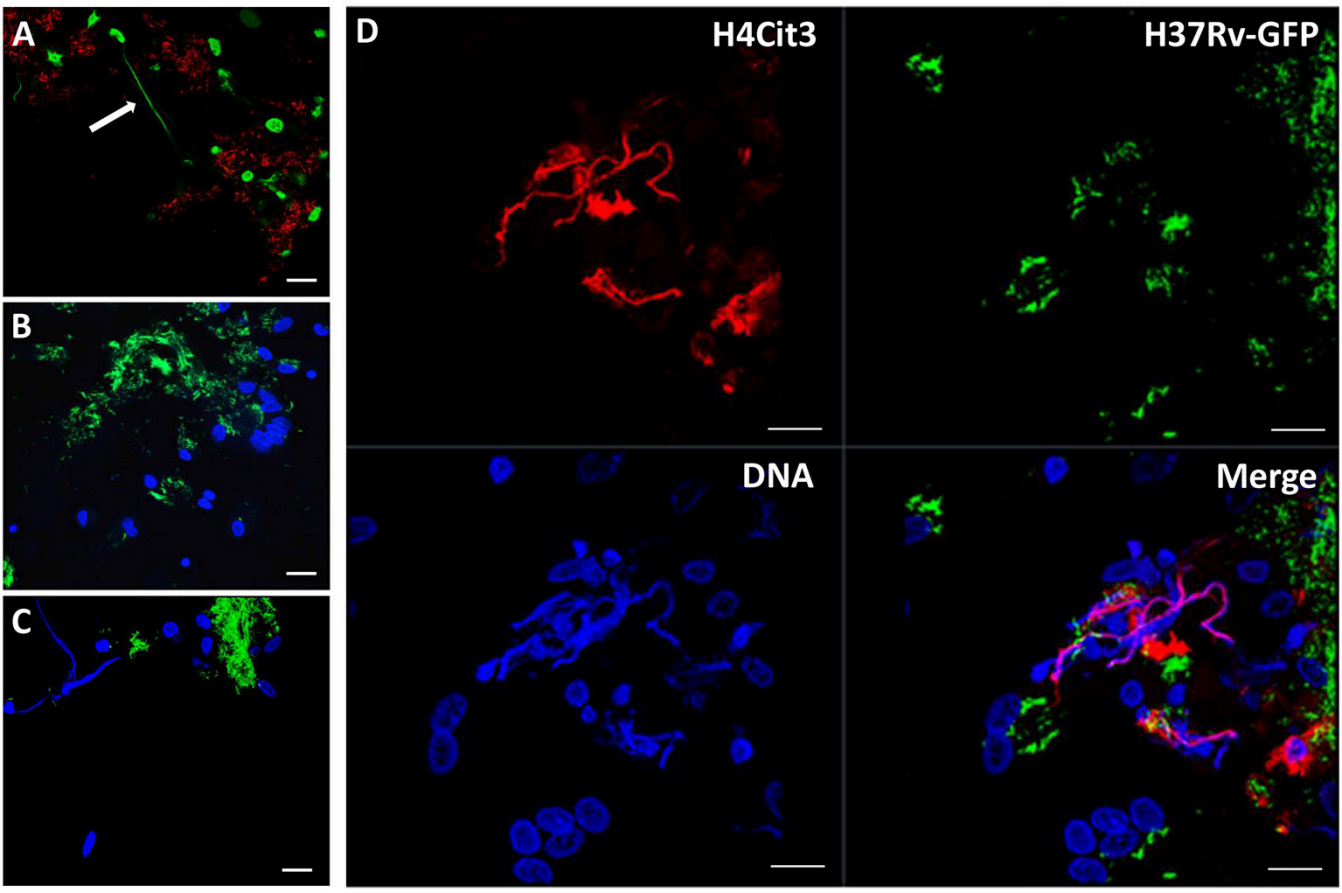
METS are composed of DNA. Macrophages were infected with the cording, **(A)** Cherry-expressing H37Rv or **(B–D)** GFP-expressing H37Rv and fixed after 24 h. DNA was stained with **(A)** picogreen or with **(B–D)** DAPI. **(B)** No METs were observed in cells treated with DNase I before fixation. **(D)** Immunofluorescence staining of citrullinated histone 4 (red) and co-staining with DAPI (blue) suggests that the DNA in METs is of nuclear origin. Scale bar: 20 μm.

**Figure 5:**
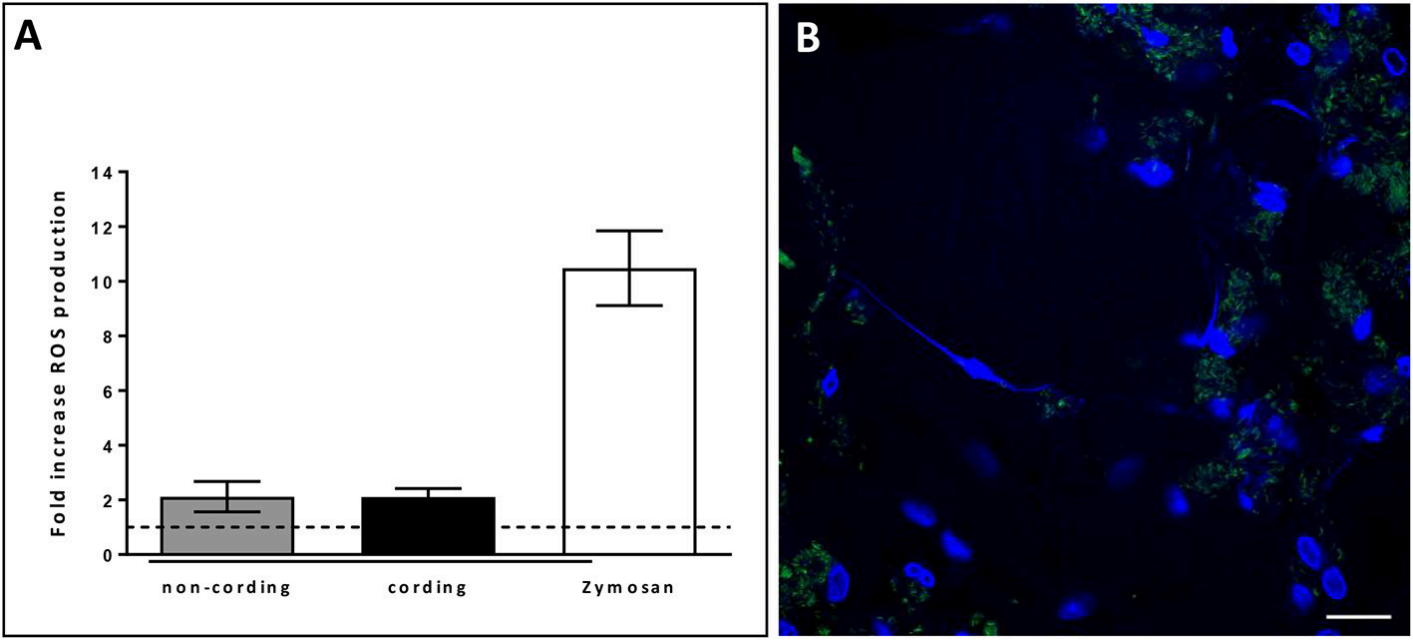
METs formation in human macrophages is independent of ROS. **(A)** Cording and non-cording bacteria induce ROS-production in macrophages to a similar extent. Zymosan was used as a positive control. Mean values ± SEM from hMDMs from 3 donors (measured in duplicates) are shown. **(B)** DPI-inhibition of ROS-production did not inhibit METs-release in hMDMs infected with the cording phenotype of H37Rv. Cells were stained with DAPI after 24 h of infection. Bar: 20 μm.

In order to verify that the observed structures were indeed METs, we performed additional experiments. First, we stained the METs triggered by cording bacteria using another dye, Picogreen, which also binds DNA (Figure 4A). This approach revealed similar structures as with DAPI (Figures 4B–D). In addition, no METs could be observed in hMDMs treated with DNase I subsequently to infection with cording bacteria (Figure 4B). ETs of nuclear origin, as opposed to ETs of mitochondrial origin, have been described to be decorated with citrullinated histones (Liu et al., 2014) and therefore we used a histone antibody (anti-H4Cit3) targeting histones to determine the origin of the METs. Confocal images showed the presence of histones that co-localized with the DAPI stain (Figure 4D). The formation of NETs by human neutrophils is dependent on the generation of ROS by NADPH oxidase (Fuchs et al., 2007; Braian et al., 2013). To determine whether ROS production is also linked to MET formation, ROS production was measured in hMDMs infected with cording or non-cording *M. tuberculosis* using luminol-enhanced chemiluminescence. Zymosan, a known inducer of ROS-production, was used as a positive control. We found that both cording and non-cording mycobacteria were very weak inducers of ROS as compared to zymosan (Figure 5A). Also, inhibition of the NADPH oxidase using diphenyleneiodonium (DPI) did not prevent MET formation (Figure 5B), suggesting that MET formation is not dependent on ROS production in hMDMs.

### Rapid intracellular growth of cording H37Rv

**Figure 6:**
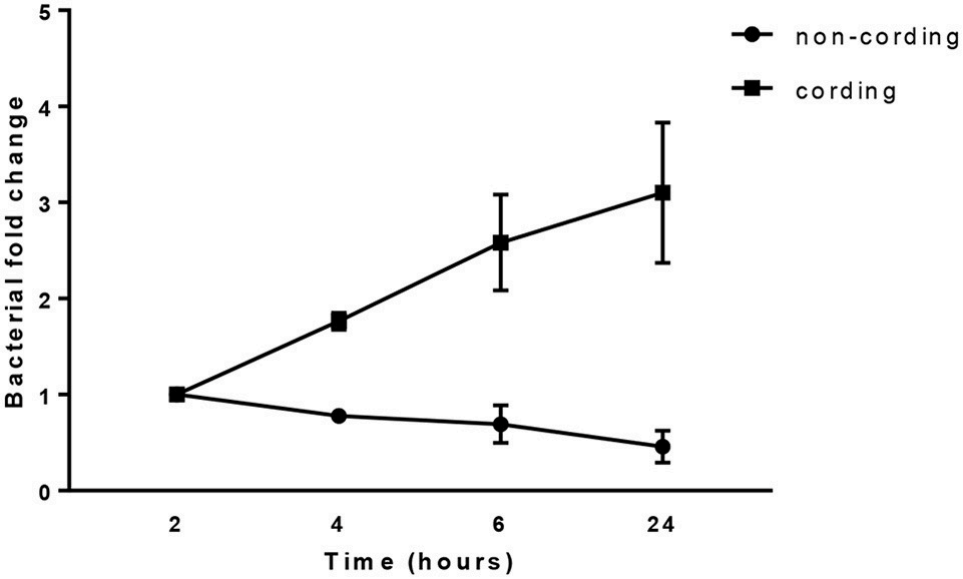
Cording *M. tuberculosis* grow rapidly during infection. hMDMs from three different donors were infected with either shaken or standing cultures of H37Rv at MOI10. The bacterial growth was measured at 2, 4, 6, and 24 h. Bacterial fold change normalized to the initial time point. The total number of bacteria was obtained by measuring luminescence in both supernatant and cell lysate after the addition of the luciferase substrate decanal.

In order to determine the replication of the bacteria during the 24 h infection of hMDMs at MOI10, we measured bacterial growth using a luminescence-based method previously established in our group (Eklund et al., 2010). The result showed a continuous growth of cording bacteria over the period as shown by an increase in bacterial load while the non-cording bacteria did not replicate in hMDMs during this time span (Figure 6). The results were not significantly different.

### ESAT-6 is essential for MET formation in *M. tuberculosis* infected macrophages

**Figure 7:**
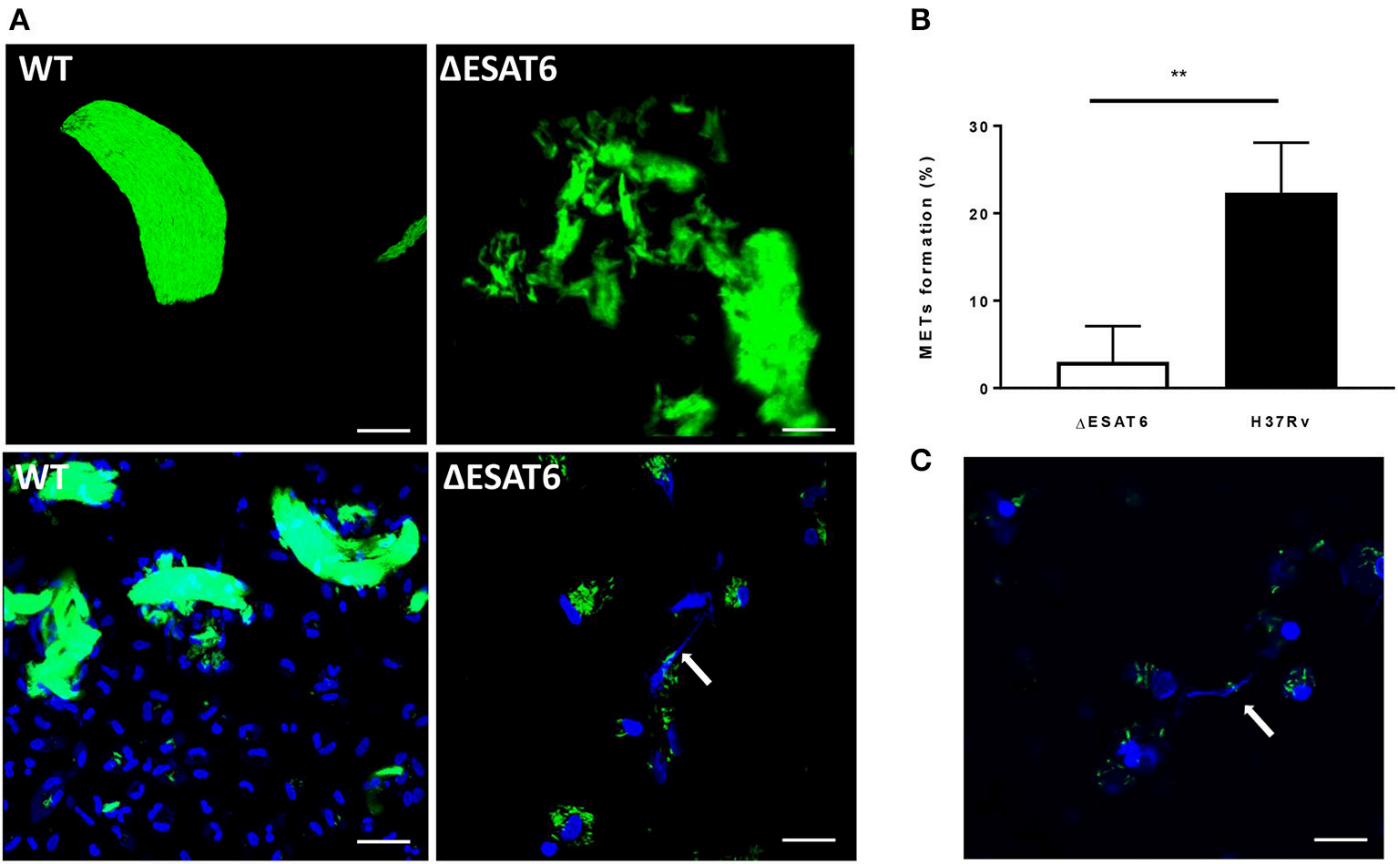
ESAT-6 is required for MET formation. **(A)** ΔESAT6 and the wild-type H37Rv strains were cultured on a shaker for 3 days with Tween_low_ and used to infect hMDMs at MOI10. The cells were fixed after 24 h and stained with DAPI to visualize DNA. Bar: 20 μm. **(B)** The percentage of cells displaying METs formation was determined using confocal microscopy *n* = 4. **(C)** hMDMs were infected with the bacteria harvested from shaken culture of *M. marinum* strain expressing GFP for 24 h, fixed and stained with DAPI. Bar: 20 μm. ^**^ *p* < 0.01.

ESAT-6 is one of the most important virulence factors that *M. tuberculosis* utilizes during interaction with human macrophages. Its membrane-lysing activity allows *M. tuberculosis* to escape out of the phagosome, kill the host cell and spread from an infected cell to other cells (Gao et al., 2004; van der Wel et al., 2007). We assessed whether ESAT-6 plays a role in *M. tuberculosis*-induced MET formation. An ESAT-6 deletion strain (ΔESAT6) and the wild-type H37Rv were grown under the cord-promoting conditions as described above. Although the ΔESAT6 also formed cords (Figure S3), those clusters were not efficient in triggering MET formation in the human macrophages (Figure 7A). Quantification of the MET formation triggered by the WT and ΔESAT6 H37Rv is shown in Figure 7B. This observation was confirmed by using another mycobacterial species *M. marinum* that can also secrete a ESAT-6 homolog (Smith et al., 2008). Like wild-type *M. tuberculosis*, the cording phenotype of *M. marinum* produced METs in hMDMs after 24 h of infection (Figure 7C).

### MET formation induced by clinical isolates

**Figure 8:**
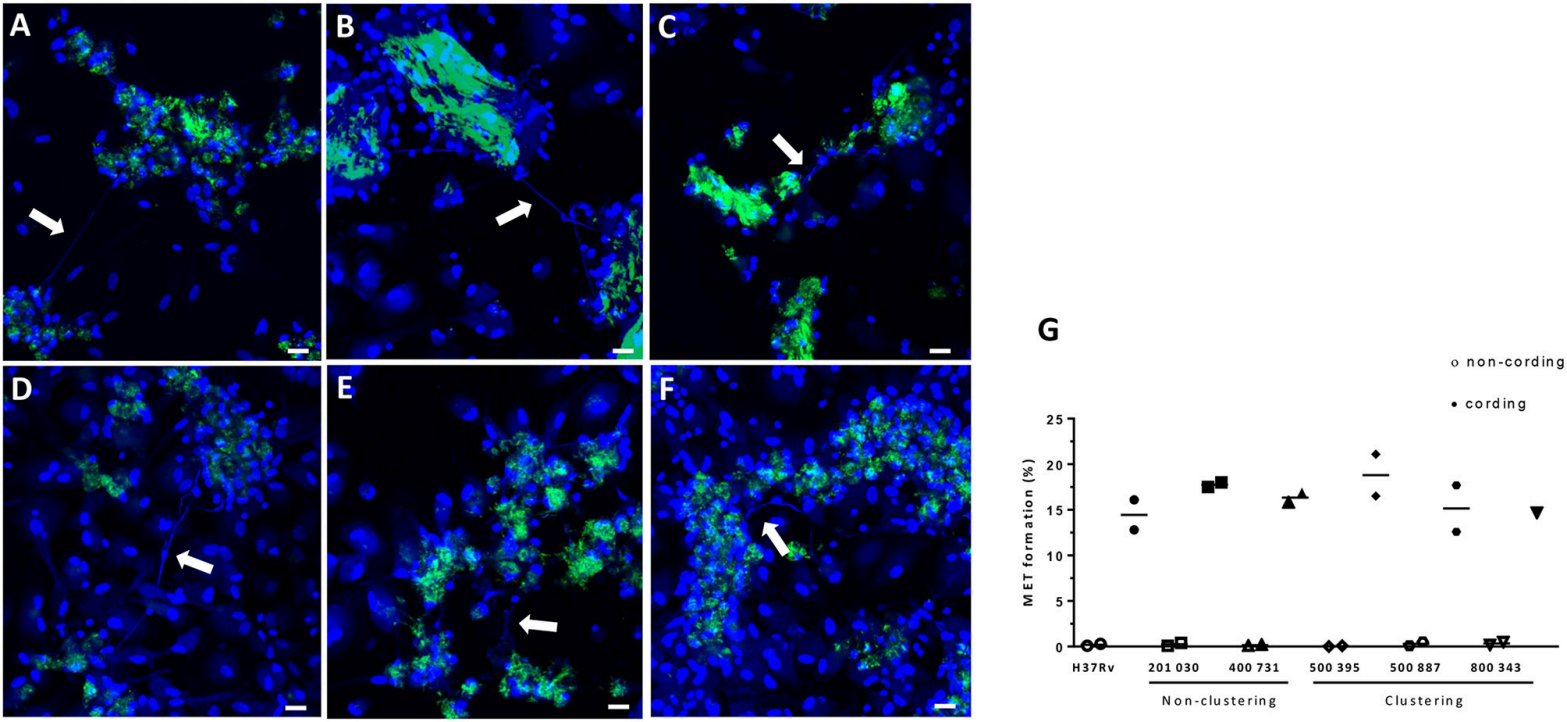
H37Rv and five clinical strains cultured under shaking conditions/Tween _low_ induce MET formation. GFP-expressing (green) *M. tuberculosis* were passaged and cultured in the presence of 0.01% Tween for 3 days on a shaker at 260 rpm. Macrophages were infected at a MOI of 10 with **(A)** H37Rv, **(B)** 201 030, **(C)** 400 731, **(D)** 500 395, **(E)** 500 887, and **(F)** 800 343. DNA was visualized with DAPI (blue). The white arrows indicate METs released the macrophages. Bar: 20 μm. **(G)** The percentage of cell nuclei producing METs 24 h after infection. Mean values of METs production are shown for each donor individually (*n* = 2).

To test the effect of cording on MET formation in other *M. tuberculosis* strains, hMDMs were infected with five clinical strains in addition to H37Rv. The chosen clinical strains were epidemiologically proven to be at the extremes of the transmission spectrum (Nebenzahl-Guimaraes et al., 2016), and therefore we were interested in testing if they differed in their ability to induce METs. All six tested strains were able to form cords when cultured on a shaker in the presence of 0.01% Tween-80 (data not shown), and all mycobacteria displaying the cording phenotype were able to induce MET formation in hMDMs (Figures 8A–F). Quantification of the percentage of MET producing nuclei 24 h after infection confirmed that cording mycobacteria induce more METs compared to non-cording mycobacteria (Figure 8G). However, the cording phenotype of the different clinical isolates were equally efficient in inducing METs.

## Discussion

Massive growth of extracellular *M. tuberculosis* has been shown to occur at the air/liquid interface in cavities in the lungs of patients with advanced, cavitary TB (Hunter et al., 2006). Like for many aerobic bacteria, oxygen is a growth rate-limiting factor for *M. tuberculosis* (Lyon et al., 1961). As expected, we found that bacteria from shaken, better oxygenated *M. tuberculosis* cultures grow faster after infection of human macrophages. The fast growth coincided with the formation of aggregates, which were demonstrated to display the organized arrangement known as the cording phenotype. To further promote aggregation/cording we also reduced the concentration of Tween-80, which is often used in *M. tuberculosis* cultures to reduce aggregation. Cord formation is a feature of *M. tuberculosis* that has been proposed to contribute to virulence (Middlebrook et al., 1947; Glickman et al., 2000). In this study, we demonstrate that the cording phenotype of *M. tuberculosis* triggers the release of METs in hMDMs. Several recent studies show that macrophages, like neutrophils, are able to release extracellular DNA in response to microbes, including mycobacteria (Jonsson et al., 2013; Wong and Jacobs, 2013; Liu et al., 2014). In support of our findings, strains of *M. abscessus* that do not form cords were readily phagocytosed, while cord-forming *M. abscessus* were surrounded by a meshwork containing DNA and histones (Jonsson et al., 2013).

The ESAT-6 secretion system ESX-1 has been identified as a major virulence factor for mycobacterial pathogenesis in previous studies (Gao et al., 2004; van der Wel et al., 2007). Given the relationship between cording morphology and virulence, we tested an ESAT-6 deletion mutant strain of *M. tuberculosis* for cord formation and the release of METs from hMDMs. Using confocal microscopy, we could observe that the ΔESAT6 also form large aggregates reminiscent of cording when cultured on a shaker. However, in contrast to the different tested wild type strains of *M. tuberculosis* and to *M. marinum*, the aggregates of ΔESAT6 *M. tuberculosis* did not induce MET formation in hMDMs. This suggests that microbe size or aggregate formation alone is not sufficient to induce MET formation. Our results support another previous finding showing that a mutant strain of *M. tuberculosis* lacking the Type VII secretion machinery (the ESX-1 locus, including ESAT-6), did not induce MET formation (Wong and Jacobs, 2013).

The release of extracellular DNA by neutrophils is dependent on ROS production by NADPH oxidase (Fuchs et al., 2007; Braian et al., 2013). However, the production of METs did not correlate with ROS production by hMDMs. Additionally, experiments with DPI (a NADPH-oxidase-dependent ROS production inhibitor) did not show an inhibiting effect on MET production by hMDMs after *M. tuberculosis* stimulation. These findings are consistent with those of other *in vitro* studies showing that MET formation is not dependent or linked to ROS and the NADPH-oxidase activity (Jonsson et al., 2013; Liu et al., 2014).

The five clinical strains of *M. tuberculosis* included in this study have been described as differing in their transmissibility (Nebenzahl-Guimaraes et al., 2016). Strains can be characterized by their molecular fingerprint and the genetic similarity among a group of strains place them in the same cluster. The strains used here that belonged to a cluster were epidemiologically considered to be at the highest extreme of the transmission spectrum and had more consistent genetic differences compared to non-clustered ones (Nebenzahl-Guimaraes et al., 2016). The strains that are not part of a cluster, did not appear to differ in their ability to induce METs compared to the clustering strains. This suggests that induction of MET formation is a common feature of cording phenotype of *M. tuberculosis* strains.

Extracellular traps have been suggested as a protective mechanism of mucosal surfaces that trap and possibly kill bacteria (Mohanty et al., 2015). The physiological relevance of METs formation in response to cording mycobacteria remains unclear. Being a conserved phenomenon that evolved before the emergence of metazoans (Zhang et al., 2016), extracellular traps may have evolved as a defense mechanism that protect social amoeba from pathogenic bacteria, including mycobacteria, which share their ecological niche. On the other hand, the observation that ESAT-6 is required for the formation of METs suggest that it is beneficial for the bacteria. As many other aspects of mycobacteria-host interaction, this phenomenon may reflect a “tug-of-war” that has been going on for billions of years. As a result, a chronic infection like human TB develops into active disease in limited parts of the lungs of some individuals, whom then can further spread the bacteria in the population for many years (Hunter et al., 2006).

In summary, we provide data showing that growing *M. tuberculosis* in broth with low concentration of Tween-80 and on a shaker promotes the formation of the cording phenotype. The cording bacteria grow very rapidly during the 24 h of infection compared to the non-cording bacteria. Further, the cording *M. tuberculosis* are able to trigger the formation of METs in an ESAT-6-dependent manner. The observed phenomenon could possibly resemble the *in vivo* situation when mycobacteria grow in lung cavities.

## Author contributions

SK: Principal investigator, data acquisition, data analysis, writing and revision of the manuscript, CB, VK: Data acquisition, data analysis, writing and revision of the manuscript, JR: Technical supervision and revision of the manuscript, ML (5th author): Supervision in scanning electron microscopy and revision of the manuscript, Rv: Revision of the manuscript, ML (7th author): Supervision of the study and revision of the manuscript.

### Conflict of interest statement

The authors declare that the research was conducted in the absence of any commercial or financial relationships that could be construed as a potential conflict of interest.
