# Supplementary material for: The Cording Phenotype of Mycobacterium tuberculosis Induces the Formation of Extracellular Traps in Human Macrophages
Source: Front Cell Infect Microbiol. 2017 Jun 26;7:278. doi: 10.3389/fcimb.2017.00278 (PMC5483443; doi:10.3389/fcimb.2017.00278)
Supplement: Supplementary file 1 [file Image1.pdf]

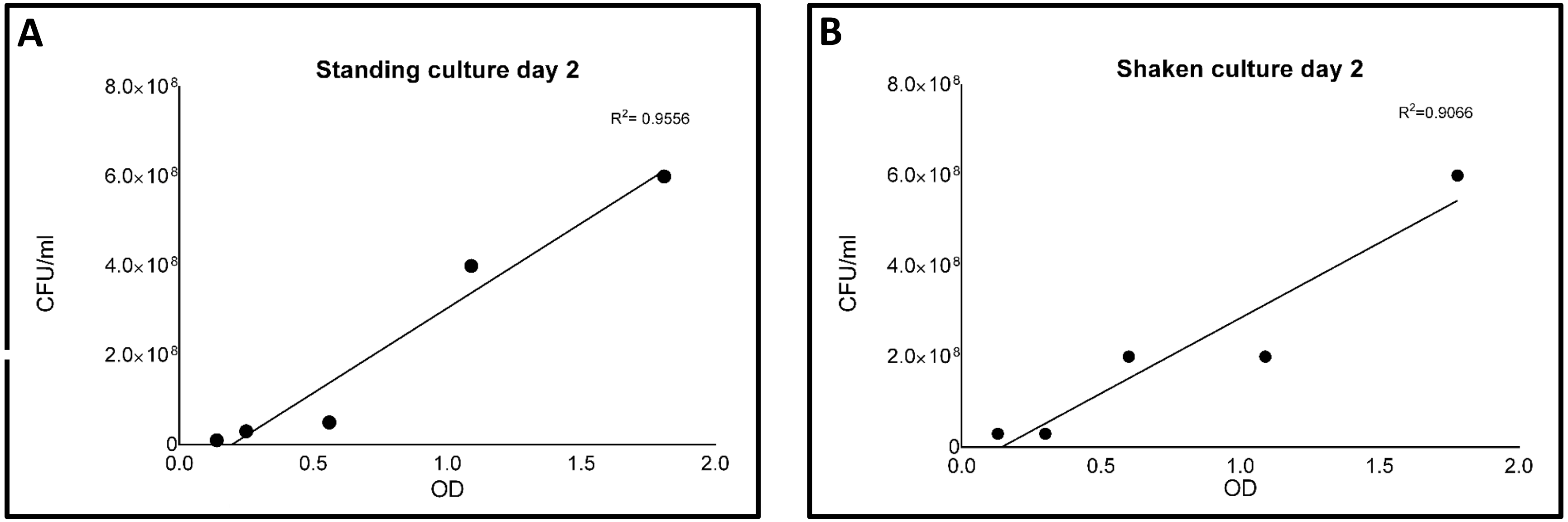

Supplemental Figure S1. Bacterial growth of **(A)** standing culture and **(B)** shaken culture of H37Rv on day 2 after reseeding. Graphs depict optical density (OD) versus CFU/ml.

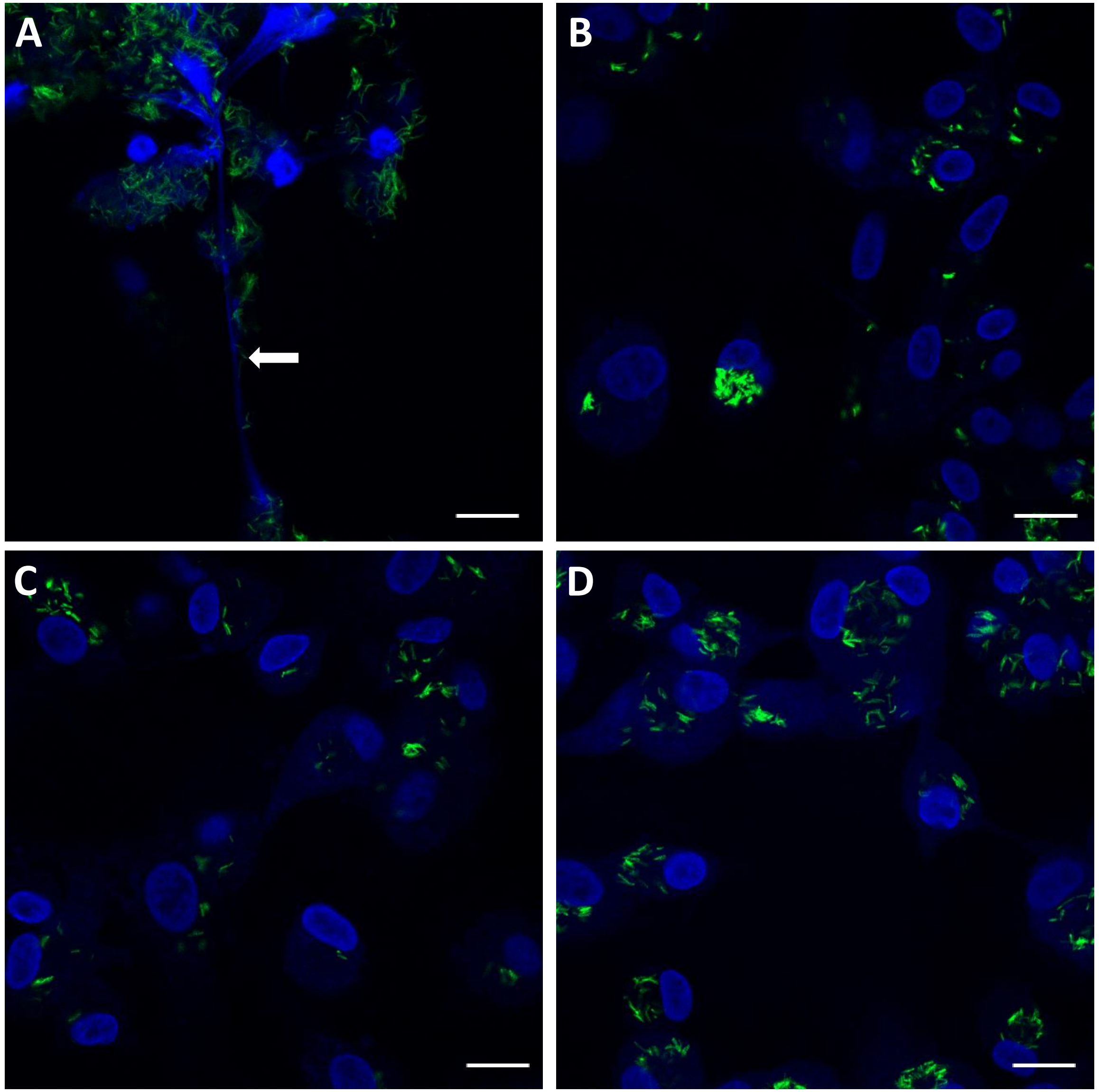

Supplemental Figure S2. Human macrophages were infected with H37Rv-GFP bacteria harvested from **(A)** shaken culture at MOI5 or with bacteria harvested from **(B)** standing culture at MOI10, **(C)** MOI20 and **(D)** MOI40. The cells were fixed after 24 h and stained with DAPI. Bar: 20µm.

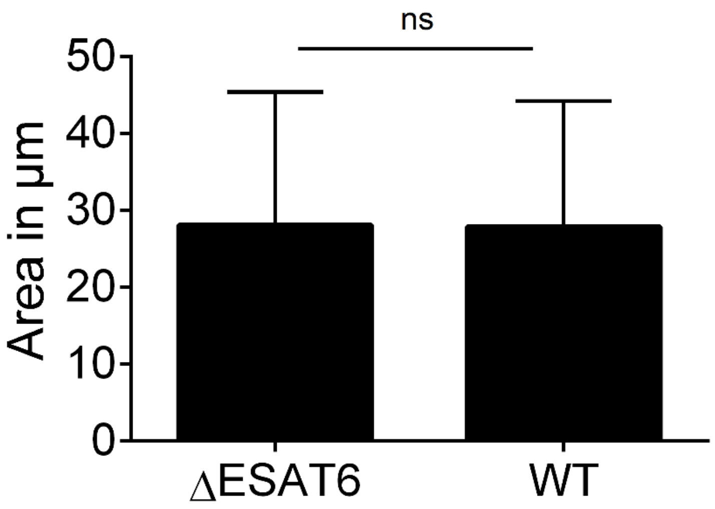

Supplemental Figure S3. Images of aggregates/cords from H37Rv-ΔESAT6 and wild-type (WT) from shaken cultures were analyzed using ImageJ, and the area of the aggregates/cords was calculated.
